# 277. A Functional OMICs Evaluation of Omadacycline to Understand Anti-*Clostridioides difficile* Protective Effects

**DOI:** 10.1093/ofid/ofad500.349

**Published:** 2023-11-27

**Authors:** Jinhee Jo, Samantha Agyapong, Chenlin Hu, Weiqun Wang, Anne J Gonzales-Luna, Chris Lancaster, Kevin W Garey

**Affiliations:** University of Houston, Houston, Texas; University of Houston, Houston, Texas; University of Houston, Houston, Texas; University of Houston College of Pharmacy, Houston, Texas; Department of Pharmacy Practice and Translational Research, University of Houston College of Pharmacy, Houston, Texas, USA, Houston, TX; University of Houston, Houston, Texas; University of Houston College of Pharmacy, Houston, Texas

## Abstract

**Background:**

Omadacycline is an aminomethylcycline tetracycline with potent *in vitro* activity against *C. difficile* (CD) and a low propensity for CD infection (CDI) in clinical trials. In addition to potent *in vitro* activity, ideal properties of anti-CDI antibiotics include favorable effects on bile acids and short-chain fatty acids (SCFA). The aim of this study was to compare bile acid and SCFA changes in healthy volunteers given oral omadacycline versus vancomycin.

**Methods:**

This was a phase 1 healthy volunteer study in 16 adults randomized to an oral 10-day course of either omadacycline or vancomycin. Stool samples were collected daily with metagenomics performed targeting the V4 region of the 16S ribosomal RNA gene using the Miseq platform (Illumina). Stool samples collected at baseline, during therapy, and follow-up visits were used for this functional omics evaluation. Targeted bile acids and SCFA quantification were performed using liquid chromatography-tandem mass spectrometry (LC-MS/MS). The final concentration of each gut metabolome was normalized by the corresponding stool sample weight.

**Results:**

Both antibiotic groups showed a significant decrease in alpha diversity during therapy. Primary bile acids increased in vancomycin-treated subjects which was not observed in the omadacycline group although secondary bile acids diminished equally. Fecal SCFA concentrations of butyrate decreased in both groups while acetate and propionate were preserved in the omadacycline group compared to vancomycin.

**Conclusion:**

Both omadacycline and vancomycin demonstrated changes in the gut microbiome with omadacycline showing favorable effects on bile acids and SCFA. These results provide mechanistic understandings on the anti-CDI properties of omadacycline.

**Disclosures:**

**Anne J. Gonzales-Luna, PharmD, BCIDP**, Cidara Therapeutics: Grant/Research Support|Ferring Pharmaceuticals: Personal Fees|Paratek Pharmaceuticals: Grant/Research Support|Seres Therapeutics: Grant/Research Support **Kevin W. Garey, MS;PharmD**, Paratek Pharmaceuticals: Grant/Research Support

